# scICER: clustering stability assessment for integrated single-cell analyses

**DOI:** 10.1093/bioadv/vbag334

**Published:** 2026-11-06

**Authors:** Beirui Yu, Donggang Xu, Zhangrui Shi, Muyang Li, Yuli Zheng, Xiaolong Cao

**Affiliations:** Department of Oncology, Zhujiang Hospital, Southern Medical University, Guangzhou 510280, China; Department of Oncology, Zhujiang Hospital, Southern Medical University, Guangzhou 510280, China; Department of Oncology, Zhujiang Hospital, Southern Medical University, Guangzhou 510280, China; Department of Oncology, Zhujiang Hospital, Southern Medical University, Guangzhou 510280, China; Department of Oncology, Zhujiang Hospital, Southern Medical University, Guangzhou 510280, China; Department of Anesthesiology, Zhujiang Hospital, Southern Medical University, Guangzhou 510280, China; Translational Medicine Research Center, Zhujiang Hospital, Southern Medical University, Guangzhou 510280, China

## Abstract

**Motivation:**

Clustering is essential in single-cell and spatial omics for identifying cell types and states, but stochastic algorithms, such as Leiden can reduce cluster stability and compromise annotation reliability. We present scICER, an R/Seurat implementation of single-cell Inconsistency Clustering Estimator (scICE)-style inconsistency analysis that evaluates clustering stability on user-provided graphs stored in Seurat objects from standard or integrated workflows.

**Results:**

Compared with the original Julia implementation, the scICER adds ecosystem-native input/output, fixed-resolution evaluation, optional handling of very small clusters, and practical workflows for large objects. Across 48 benchmark datasets and integrated analyses, scICER reproduced reference scICE behavior while enabling stability assessment after Harmony or scVI correction.

**Availability and implementation:**

scICER is implemented in R and is freely available at https://github.com/ATPs/scICER. A companion Python implementation with similar functionality, scICEpy, is available at https://github.com/ATPs/scICEpy.

## 1 Introduction

The rapid development of single-cell and spatial omics technologies, including scRNA-seq (Hwang *et al.* 2018), scATAC-seq (Shi *et al.* 2022), and spatial transcriptomics (Gulati *et al.* 2025), has enabled high-resolution profiling of individual cells and their spatial context. Across these platforms, clustering is a critical step for grouping cells by molecular similarity to define cell types, subtypes, or transient states (Kiselev *et al.* 2019, Van den Berge *et al.* 2020, Jin *et al.* 2021), forming the basis for downstream analyses such as differential expression, trajectory inference, and cell–cell communication.

Graph-based methods, particularly the Leiden algorithm, are widely used due to their efficiency and ability to detect well-connected communities (Luecken and Theis 2019). However, their stochastic optimization can produce inconsistent clustering results across runs, as random node ordering may alter partitions and even cause clusters to appear or disappear (Traag *et al.* 2019).

To address this, single-cell Inconsistency Clustering Estimator (scICE) was developed to evaluate clustering stability across multiple runs and identify reliable cluster numbers (Kim *et al.* 2025). However, the original Julia implementation is not directly embedded in the two most common single-cell analysis ecosystems: Seurat (Hao *et al.* 2024) in R and AnnData/Scanpy (Wolf *et al.* 2018) in Python. Moreover, modern studies frequently integrate multiple samples using Harmony (Korsunsky *et al.* 2019) or scVI (Lopez *et al.* 2018), generating corrected embeddings for clustering, yet scICE does not support these workflows.

We developed scICER to make scICE-style clustering stability assessment accessible within Seurat workflows. scICER operates directly on Seurat graphs and adds practical features for integrated analyses, including manual resolution evaluation and optional handling of very small clusters. A companion Python implementation, scICEpy, provides similar functionality for AnnData workflows but is presented here as a secondary implementation.

## 2 Implementation

scICER accepts a Seurat object and clusters a specified graph stored in its graph slot (object@graphs), including shared nearest-neighbor (SNN), *k*-nearest-neighbor (KNN), or Uniform Manifold Approximation and Projection (UMAP)-derived graphs generated from principal component analysis, Harmony, scVI, scLENS (Lopez *et al.* 2018, Korsunsky *et al.* 2019, Kim *et al.* 2024), or other reductions. Other R object classes, such as SingleCellExperiment, are not directly supported. Users can evaluate clustering stability either across a prespecified range of cluster numbers or at user-defined Leiden resolution values. In addition to reproducing the core scICE framework, scICER supports manual testing of selected resolutions, reproducible analyses through fixed random seeds, and reporting based on the final cluster labels returned by the analysis. scICEpy implements a similar interface for AnnData objects and can be used by Python users who prefer Scanpy-compatible workflows. The benchmark analyses presented in this study focus on scICER, while scICEpy provides the corresponding Python implementation for AnnData-based workflows.

scICER builds on the concept of multiple runs of Leiden clustering originally used in scICE (Kim *et al.* 2025), but it supports two analysis modes. In cluster-range mode, “cluster_range” specifies target cluster numbers *K*, which are not passed directly to Leiden. Instead, scICER performs a shared coarse-to-fine sweep over Leiden resolution values, records the number of clusters returned at each resolution, and uses this resolution–cluster-count trajectory to identify intervals corresponding to the requested *K* values. Additional resolution values are evaluated for unresolved targets as needed, after which candidate resolutions within each interval are assessed using repeated Leiden runs and the most consistent candidate is retained for stability assessment. In manual-resolution mode, scICER skips the target-*K* search and evaluates the user-provided resolution values directly, while still repeating Leiden clustering for each resolution. When a seed is provided, scICER uses deterministic seeding to make the repeated runs reproducible while preserving stochastic variation across trials. Agreement among labelings is quantified by element-centric similarity (ECS; Gates *et al.* 2019). ECS measures cell-level agreement between two clustering partitions by comparing the cluster–membership relationships of each cell, and the mean ECS across cells represents the overall similarity between the two partitions. ECS is computed with the “element_sim” function from ClustAssess (Shahsavari *et al.* 2022). Following the original scICE framework (Kim *et al.* 2025), repeated Leiden clustering outcomes are summarized using the inconsistency coefficient (IC), defined as the reciprocal of the expected ECS; IC = 1 indicates perfect consistency, whereas larger values indicate increasing inconsistency. Bootstrap resampling is used to estimate stability, and stable cluster numbers are reported under a user-defined threshold (1.005 by default). For benchmark analyses, the consistent clustering ratio was defined as the number of distinct stable *K* values divided by 19, corresponding to the requested range *K* = 2–20.

Compared with the original Julia implementation, scICER adds practical controls for Seurat workflows while preserving the same stability-assessment principle. Users can evaluate either a range of target cluster numbers or specific Leiden resolution values, return the selected labels directly to the Seurat object, and optionally merge very small final clusters for cleaner downstream annotation. These features make scICER easier to use in integrated analyses while keeping the stability assessment consistent with the original scICE concept. For large objects, scICER can run on a reduced Seurat object containing only the graph and minimal metadata before merging labels back to the original object. scICEpy provides analogous lightweight H5AD workflows. These designs reduce unnecessary assay-memory use during stability assessment.

To assess implementation fidelity, we benchmarked scICER against the original scICE analyses on the same 48 real and simulated datasets used by Kim *et al.* (2025). These datasets span ∼700 to >12 000 cells and include both hierarchical and nonhierarchical structures. Following the scICE benchmark, we evaluated *K* = 2–20, excluding *K* = 1 as a trivially stable, uninformative solution. This range is benchmark-specific; scICER allows users to specify other *K* ranges or Leiden resolutions. We first performed direct comparisons on matched scLENS-derived embeddings and UMAP graphs (Kim *et al.* 2024). We then tested a native R workflow in which the UMAP graph was regenerated with “uwot” (version 0.2.3) using minimum distance 0.1, cosine metric, and 15 nearest neighbors. SNN and KNN graphs were constructed with Seurat “FindNeighbors” using Euclidean distance and 20 nearest neighbors.

We further evaluated three preprocessing workflows, log-normalized RNA, SCTransform, and scLENS, combined with three graph types, SNN, KNN, and UMAP. To demonstrate integration compatibility, we applied scICER to the human pancreas dataset from the atlas-level integration benchmark (Luecken *et al.* 2022) after Harmony or scVI correction within Seurat (Lopez *et al.* 2018, Korsunsky *et al.* 2019). We also applied scICER to a separate pancreatic islet atlas (GSE221156; 245 878 cells) as a larger real-data use case. For scVI, highly variable genes were used as input features, and in both integration workflows stability was evaluated on UMAP graphs derived from the corrected embeddings. Performance was summarized only as a practical benchmark on a computing node equipped with dual AMD EPYC 9754 processors and 1 TB RAM.

## 3 Results

### 3.1 Fidelity to the reference scICE workflow

scICER reproduced the reference scICE behavior with high concordance across the 48 benchmark datasets (Kim *et al.* 2025). On matched scLENS-derived inputs, scICER and reproduced scICE selected the same optimal cluster number for 44 of 48 datasets, while the complete consistent cluster set was identical for 23 of 48 datasets (Fig. 1, available as supplementary data at *Bioinformatics Advances* online). The mean stable-set Jaccard overlap was 0.895 (median, 0.917; range, 0.455–1.000; Table 1, available as supplementary data at *Bioinformatics Advances* online). Overall and hierarchy-stratified concordance summaries support this conclusion (Fig. 1A and B), while full per-dataset and scICE-reproduction comparisons are retained separately (Fig. 1 and 2A and B, available as supplementary data at *Bioinformatics Advances* online). In our reproduction, scICER placed the optimal label within the consistent cluster set for 44 of 48 datasets, compared with 41 of 48 for scICE. These results indicate stronger agreement in the principal cluster-number selection than in the exact boundaries of the consistent cluster sets.

**Figure 1:**
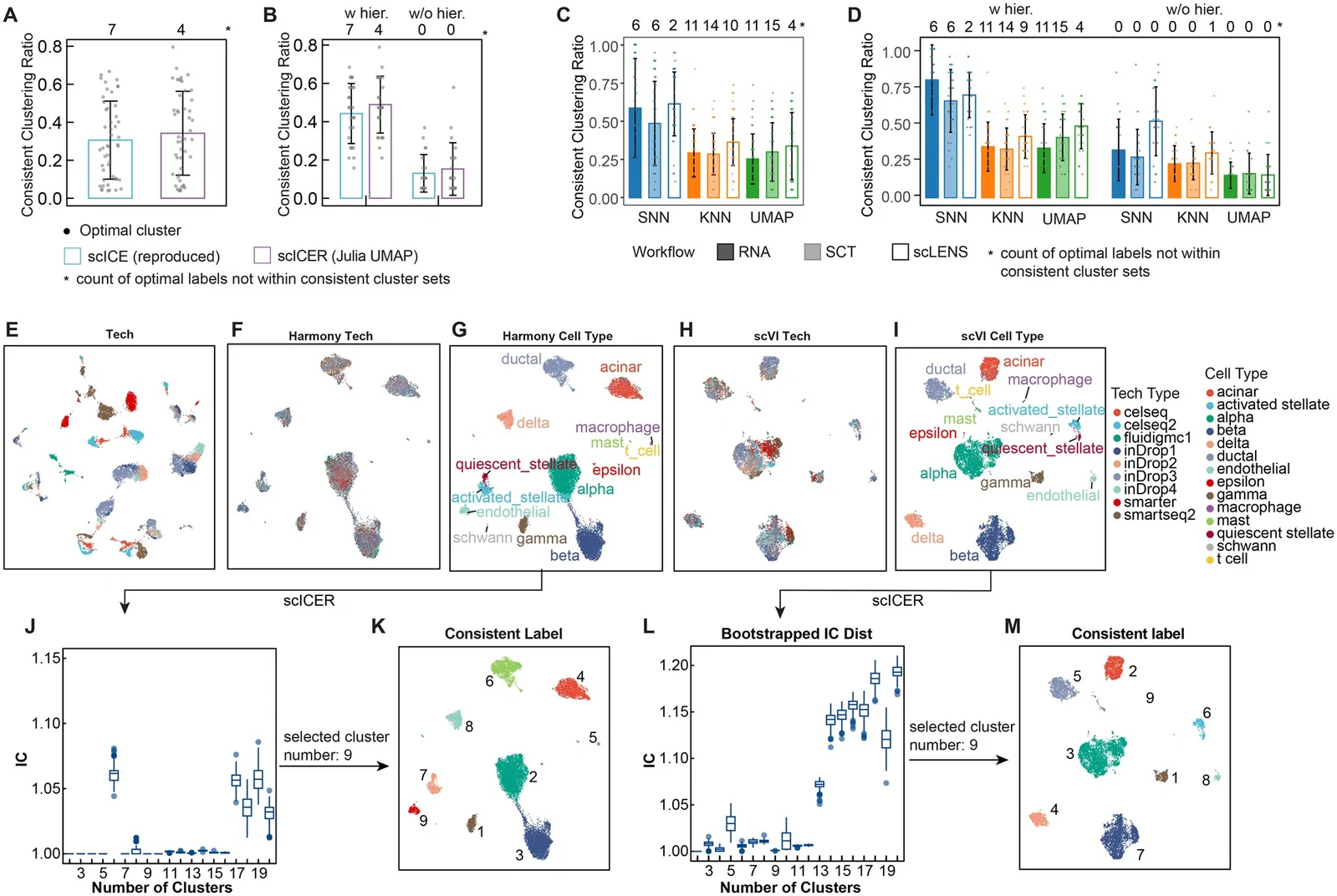
scICER validation summary and application to an integrated human pancreas dataset. (A, B) Consistent clustering ratios across 48 benchmark datasets for scICE run directly in Julia on the scLENS-derived UMAP graph [scICE (reproduced)] and scICER run on the same graph [scICER (Julia UMAP)]. The consistent clustering ratio is defined as the number of consistent cluster numbers divided by the 19 requested values (*K* = 2–20), with cluster numbers considered consistent when the median bootstrapped IC was ≤1.005. Results are shown for all datasets (A) and for datasets stratified by the presence or absence of hierarchical structure (B). Grey dots represent individual datasets, bar plots summarize distributions, and numbers above plots indicate datasets in which the optimal cluster label was not included in the consistent cluster set. (C, D) Consistent clustering ratios across nine analysis combinations generated from three preprocessing workflows (log-normalized RNA, SCTransform, and scLENS) and three graph construction methods (SNN, KNN, and UMAP). Results are shown for all datasets (C) and for datasets stratified by hierarchical structure (D). The optimal cluster number was defined as the *K* with the highest ECS to the reference labels; numbers above A–D indicate datasets in which this *K* was not included in the corresponding consistent cluster set. (E) UMAP of the uncorrected human pancreas dataset colored by sequencing technology. (F,G) Harmony-corrected embeddings colored by sequencing technology (F) or annotated cell type (G). (H, I) scVI-corrected embeddings colored by sequencing technology (H) or annotated cell type (I). (J, L) Bootstrapped IC distributions across candidate cluster numbers for Harmony-corrected (J) and scVI-corrected (L) analyses. (K, M) Stable clustering labels corresponding to the selected solutions for Harmony (K) and scVI (M) workflows. In both integration analyses, the optimal solution was obtained at *K* = 9. For the integrated pancreas analyses, *K* = 9 was selected by the highest ECS to the annotated cell types among candidates meeting the median bootstrapped IC threshold.

To evaluate the robustness of scICER in R analysis scenarios, we compared two analysis strategies: using a precomputed scLENS UMAP graph versus generating a UMAP graph *de novo* within R. The Seurat package does not include a function to generate a UMAP graph; we generated the UMAP graph with custom scripts. Despite technical differences between UMAP implementations, both approaches yielded highly consistent results (Fig. 2C–F, available as supplementary data at *Bioinformatics Advances* online), confirming scICER’s independence from specific upstream tools and seamless integration into standard R workflows.

### 3.2 Stability depends on preprocessing and graph construction

Across nine workflow–graph combinations, stable cluster ranges depended on both preprocessing (log-normalized RNA, SCTransform, and scLENS) and graph construction (SNN, KNN, and UMAP; Fig. 1C and D; Fig. 3, available as supplementary data at *Bioinformatics Advances* online). SNN-based workflows generally produced broader sets of stable clusters. This pattern may partly reflect the shared-neighbor construction of SNN graphs, but it cannot be attributed to graph type alone because the comparisons also differed in parameter settings: SNN/KNN graphs used Euclidean distance with 20 neighbors, while UMAP graphs employed cosine distance with 15 neighbors and different topological constraints (Fig. 3, available as supplementary data at *Bioinformatics Advances* online). Under these tested settings, SNN-based workflows, therefore, showed higher consistent clustering ratios than KNN- and UMAP-based workflows across all datasets (Fig. 1C). Dataset structure also influenced results: hierarchical datasets more often produced larger stable sets, especially with SNN and UMAP (Fig. 1D). scLENS + SNN yielded the highest consistent clustering ratio under the tested settings, with the optimal cluster number falling outside the consistent cluster set in only two datasets, compared with four for scLENS + UMAP. All nine workflow–graph combinations showed broadly similar patterns on nonhierarchical datasets, while scLENS minimized cases where the optimal cluster number fell outside the consistent clustering sets across graph methods.

These patterns require cautious interpretation: broader stable ranges indicate greater clustering reproducibility but not necessarily better biological resolution, because stable solutions may still merge related or locally distinct populations. For the benchmark datasets, the optimal cluster number was defined independently of the stability criterion as the *K* with the highest ECS to the ground-truth labels, and its inclusion within the corresponding consistent cluster set was evaluated across workflow–graph combinations (Fig. 3, available as supplementary data at *Bioinformatics Advances* online). We, therefore, view scICER as a tool for narrowing the candidate *K* range rather than selecting a final resolution automatically, which still requires validation using marker coherence, reference annotations, population preservation, and biological context.

### 3.3 Iterative refinement of underresolved clusters in the scICER workflow

Single-resolution clustering can lead to underclustering when algorithms prioritize global stability over local structure resolution. We demonstrated a Seurat-native iterative refinement workflow using scICER with two benchmark datasets, ZhengMix_4757 and sim_Tcell_2.

Initial scICER analysis identified six stable clusters in ZhengMix_4757 and 10 in sim_Tcell_2 (Fig. 4A–F, available as supplementary data at *Bioinformatics Advances* online), but ground truth labels revealed underclustering, with multiple T-cell subtypes merged into single clusters. Reapplying scICER to isolated merged clusters resolved this heterogeneity, identifying two and three stable subclusters, respectively. Consequently, total clusters increased from 6 to 7 in ZhengMix_4757 and from 10 to 12 in sim_Tcell_2, improving concordance with known cellular compositions (Fig. 4G–L, available as supplementary data at *Bioinformatics Advances* online). These results demonstrate that scICER can support iterative refinement of underresolved clusters within a Seurat workflow.

### 3.4 scICER enables robust analysis of integrated datasets

scICER enables robust analysis of integrated datasets, overcoming a key limitation of scICE, its incompatibility with widely used R-based integration workflows. Using a human pancreatic scRNA-seq dataset with substantial batch effects, we evaluated Harmony and scVI integration (Luecken *et al.* 2022). In the original data, cells clustered by sequencing technology rather than biological identity (Fig. 1E; Fig. 5A and B, available as supplementary data at *Bioinformatics Advances* online). scLENS produced more compact clusters but failed to remove batch effects (Fig. 5C and D, available as supplementary data at *Bioinformatics Advances* online). In contrast, Harmony and scVI reduced technology-associated separation while preserving recognizable annotated cell populations (Fig. 1F–I). scICER was subsequently applied to graphs derived from the integrated representations to assess clustering stability. Among consistent cluster numbers (median IC ≤ 1.005), *K* = 9 achieved the highest ECS to annotated cell types in both Harmony (0.9029) and scVI (0.9082) analyses **(**Fig. 1J–M; Table 2, available as supplementary data at *Bioinformatics Advances* online**).** This annotation-informed selection was used for validation of the integrated benchmark rather than as an automatic cluster-number determination by scICER. Cell-type-to-cluster correspondence of the *K* = 9 solutions was further assessed using contingency heatmaps for the Harmony and scVI analyses (Fig. 5E, available as supplementary data at *Bioinformatics Advances* online). Integration improved local batch mixing, but rare cell types were not cleanly separated: across the five types with <1% abundance, macroprecision was low despite high recall (Harmony, 18.5% and 92.3%; scVI, 21.1% and 96.0%), while normalized 30-neighbor technology entropy increased from 0.370 to 0.685 and from 0.370 to 0.554, respectively (Table 3, available as supplementary data at *Bioinformatics Advances* online). Similar stable results were also observed in a larger pancreatic islet atlas comprising 245 878 cells from GSE221156, further supporting the applicability of scICER to substantially larger real-world datasets (Fig. 6A–C, available as supplementary data at *Bioinformatics Advances* online).

### 3.5 Practical performance benchmark

We report computational performance as practical benchmarks. Across 1–40 configured workers, scICER generally traded shorter wall-clock time for higher memory use relative to scICE (Fig. 7, available as supplementary data at *Bioinformatics Advances* online). Input dimensions, graph characteristics, and core-function input object sizes are reported for each dataset and implementation (Table 4, available as supplementary data at *Bioinformatics Advances* online). On a dataset of ∼12 000 cells, at the 40-worker setting, scICER completed the clustering-stability analysis in 120 s versus 706 s for scICE (Fig. 7A, available as supplementary data at *Bioinformatics Advances* online), corresponding to an ∼ 5.9-fold speedup. This was accompanied by higher CPU utilization (Fig. 7B, available as supplementary data at *Bioinformatics Advances* online), whereas peak memory was slightly lower for scICER (17.05 versus 19.89 GB; Fig. 7C, available as supplementary data at *Bioinformatics Advances* online). Despite the higher memory demand, this trade-off remains practical for standard bioinformatics servers, and the memory requirement can be further mitigated by reducing the input Seurat object. For the GSE221156 pancreatic islet atlas of 245 878 cells, using a reduced Seurat object retaining the *harmony_snn* graph reduced peak memory usage from 50.8 to 6.2 GB at *t *= 5 and *K *= 12, corresponding to an 87.8% reduction (Fig. 7D, available as supplementary data at *Bioinformatics Advances* online).

## 4 Discussion

The stochasticity of graph-based clustering remains a major challenge for reproducible single-cell and spatial omics analysis, particularly in integrated datasets shaped by multiple sources of batch variation. Although scICE provides an elegant framework for evaluating clustering stability, its Julia implementation limits accessibility for the predominantly R-based single-cell community. scICER extends scICE-style clustering stability assessment into the R/Seurat ecosystem, which allows users to evaluate stability on the same graphs used for downstream annotation, including graphs derived from integrated embeddings. This compatibility allows clustering stability to be assessed within established workflows, thereby improving reproducibility and confidence in biological interpretation. scICEpy provides a companion Python interface with similar functionality for AnnData users, enabling similar stability-assessment workflows in Python-based single-cell analyses.

The tool should be interpreted as a stability assessment framework rather than an automatic cell-type annotation method. While a stable cluster number does not necessarily imply optimal biological resolution, scICER can help guide users toward biologically meaningful clustering. Final cluster selection should combine scICER outputs with marker coherence, batch mixing, rare-cell preservation, and biological knowledge.

scICER currently focuses on Leiden clustering, following the original scICE framework. Other community detection algorithms may exhibit different stability behaviors, and robin2 provides a complementary framework for evaluating robustness across multiple algorithms under network perturbation (Policastro *et al.* 2025). Although spatial transcriptomics was not directly benchmarked in this study, scICER may be applicable to spatial workflows in the future when an appropriate graph is available in a compatible Seurat object. Overall, scICER provides a validated and efficient framework for rigorous stability assessment in single-cell studies and could potentially support spatial analyses as well.

## Supplementary Material

vbag334_Supplementary_Data

## Data Availability

scICER is available at https://github.com/ATPs/scICER, and the companion Python implementation scICEpy is at https://github.com/ATPs/scICEpy. The scICER workflows, data preprocessing, and visualization code are available at https://github.com/ATPs/scICER-workflow. The 48 benchmark datasets were obtained from the original scICE study (https://zenodo.org/records/15113898). The human pancreas integration benchmark dataset is available at https://doi.org/10.6084/m9.figshare.12420968, and the pancreatic islet atlas is available under GEO accession GSE221156.
