# Supplementary material for: scICER: clustering stability assessment for integrated single-cell analyses
Source: Bioinform Adv. 2026 Nov 6;6(1):vbag334. doi: 10.1093/bioadv/vbag334 (PMC13770477; doi:10.1093/bioadv/vbag334)
Supplement: Supplementary file 1 [file vbag334_supplementary_data.zip › Supplementary Figures.pdf]

Fig. S1

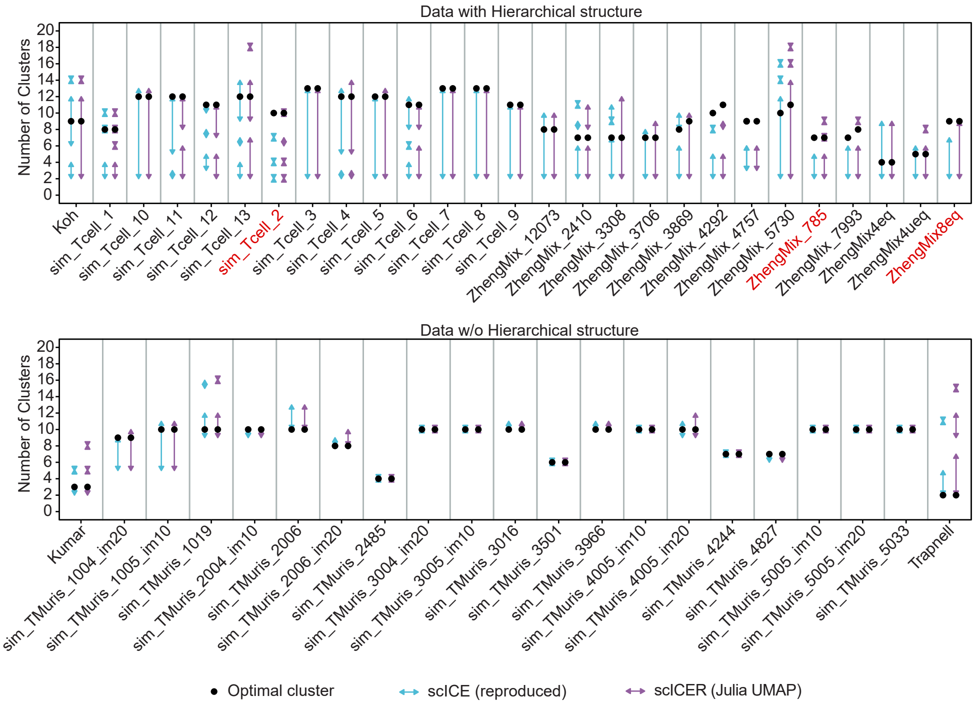

Fig. S1 Per-dataset comparison of consistent cluster sets identified by scICE and scICER. Algorithmic fidelity was evaluated across the 48 benchmark datasets from the original scICE study using the strictest matched-input design, in which the same scLENS-derived UMAP graph was supplied to both implementations. In these benchmark runs, candidate cluster numbers from 2 to 20 were evaluated in cluster_range mode by locating resolution intervals that reproduced each target cluster number, repeating Leiden clustering across admissible resolutions, and summarizing reproducibility with the bootstrapped Inconsistency Coefficient (IC). Cluster numbers with median bootstrapped IC below 1.005 formed the consistent cluster set. Within that stable set, the benchmark optimal cluster number was chosen by the highest Element-Centric Similarity (ECS) to the reference labels, so IC quantifies reproducibility whereas ECS determines which reproducible solution best agrees with the known cell-type structure. scICE (reproduced) denotes the original Julia workflow rerun on the reference UMAP graph, whereas scICER (Julia UMAP) denotes the R/Seurat implementation applied to that same imported graph so that the only substantive difference lies in the implementation of the clustering-stability analysis itself. Hierarchical datasets occupy the upper block and non-hierarchical datasets the lower block. For each dataset, the colored vertical interval marks the consistent cluster set, the black dot marks the ECS-selected optimal cluster number, and red dataset names identify cases in which the two methods differ in whether that optimal cluster number is retained within the stable set. Under these matched-input conditions, the two implementations recovered the same optimal cluster number for 44 of 48 datasets and the same full stable interval for 23 datasets. Concordance was complete across the non-hierarchical subset, whereas the remaining disagreements were confined to hierarchical datasets, in which partially nested cell populations permit several adjacent resolutions to remain simultaneously stable. The optimal cluster number remained inside the consistent set for 44 datasets in scICER (Julia UMAP) and 41 datasets in scICE (reproduced), indicating that interval boundaries were somewhat more sensitive than the principal cluster-selection result.

Fig. S2

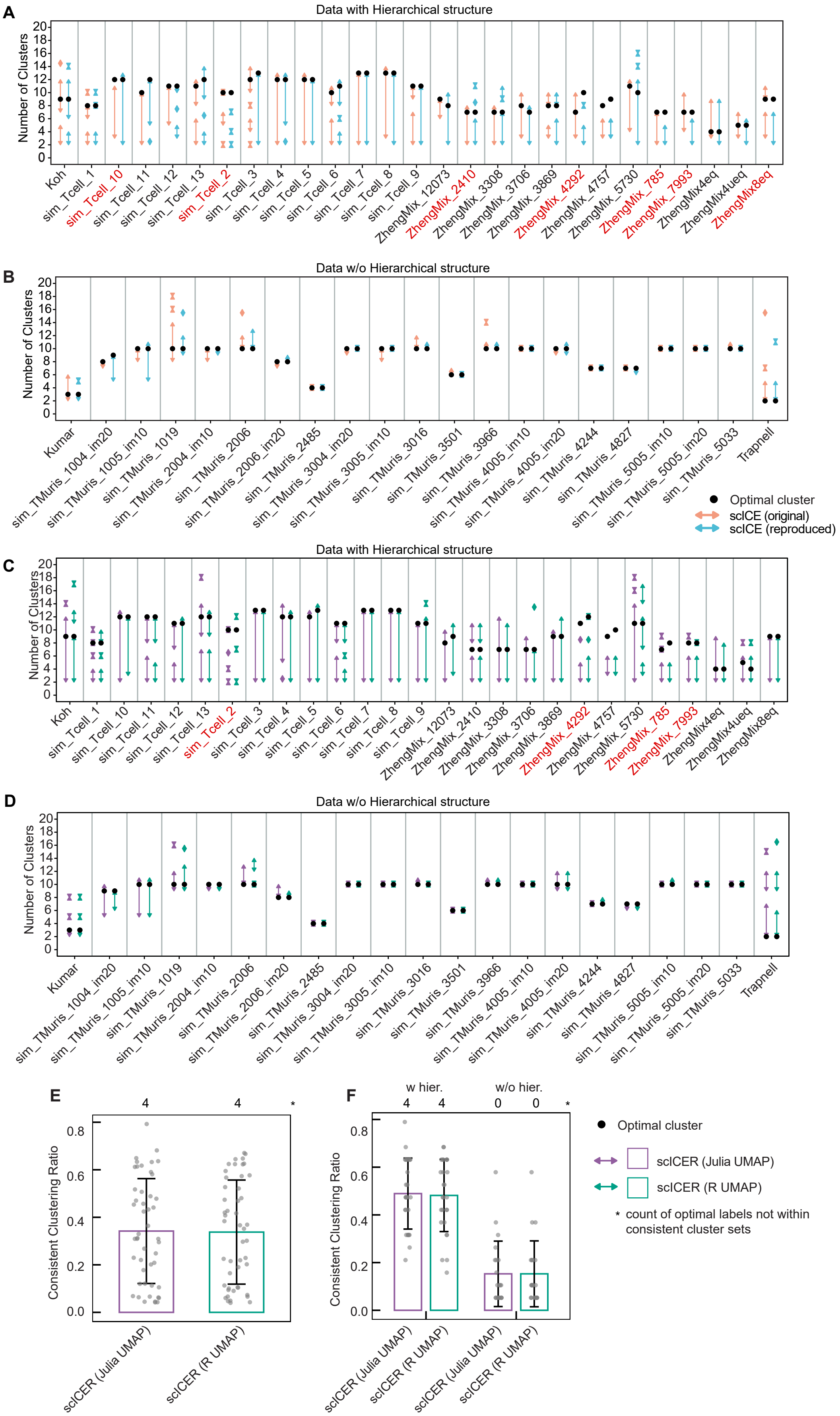
A
Data with Hierarchical structure
Number of Clusters
Koh
sim_Tcell_1
sim_Tcell_10
sim_Tcell_11
sim_Tcell_12
sim_Tcell_13
sim_Tcell_2
sim_Tcell_3
sim_Tcell_4
sim_Tcell_5
sim_Tcell_6
sim_Tcell_7
sim_Tcell_8
sim_Tcell_9
ZhengMix_12073
ZhengMix_2410
ZhengMix_3308
ZhengMix_3706
ZhengMix_3869
ZhengMix_4292
ZhengMix_4757
ZhengMix_5730
ZhengMix_785
ZhengMix_7993
ZhengMix4eq
ZhengMix4ueq
ZhengMix8eq
B
Data w/o Hierarchical structure
Number of Clusters
Kumar
sim_TMuris_1004_im20
sim_TMuris_1005_im10
sim_TMuris_1019
sim_TMuris_2004_im10
sim_TMuris_2006
sim_TMuris_2006_im20
sim_TMuris_2485
sim_TMuris_3004_im20
sim_TMuris_3005_im10
sim_TMuris_3016
sim_TMuris_3501
sim_TMuris_3966
sim_TMuris_4005_im10
sim_TMuris_4005_im20
sim_TMuris_4244
sim_TMuris_4827
sim_TMuris_5005_im10
sim_TMuris_5005_im20
sim_TMuris_5033
Trapnell
Optimal cluster
scICE (original)
scICE (reproduced)
C
Data with Hierarchical structure
Number of Clusters
D
Data w/o Hierarchical structure
Number of Clusters
E
Consistent Clustering Ratio
4
4
*
scICER (Julia UMAP)
scICER (R UMAP)
F
w hier.
w/o hier.
4
4
0
0
*
Consistent Clustering Ratio
scICER (Julia UMAP)
scICER (R UMAP)
scICER (Julia UMAP)
scICER (R UMAP)
Optimal cluster
scICER (Julia UMAP)
scICER (R UMAP)
* count of optimal labels not within consistent cluster sets

Fig. S2 Reproducibility of published scICE results and robustness to R-based UMAP-graph reconstruction. Two sources of variation are disentangled here: divergence introduced when reproducing the published scICE benchmark and divergence introduced when the UMAP graph is rebuilt natively within an R workflow. Published and reproduced scICE results are contrasted separately for hierarchical and non-hierarchical datasets (**A, B**). scICE (original) denotes the consistent cluster sets and optimal labels reported in the published study, and scICE (reproduced) denotes the Julia workflow rerun locally on the same scLENS-derived UMAP graph used for the matched-input benchmark. Colored intervals and black dots retain the definitions used in **Fig. S1**, and red dataset names again identify cases in which the ECS-selected optimal cluster number falls outside the corresponding consistent set. Agreement between the published and reproduced analyses was obtained for 38 of 48 optimal cluster numbers, whereas exact agreement of the full stable interval was limited to 7 datasets, indicating that much of the interval-level divergence arose before any comparison between Julia and R implementations. The second comparison addresses graph-construction robustness within scICER itself. scICER (Julia UMAP) uses the imported scLENS-derived UMAP graph, whereas scICER (R UMAP) uses a graph reconstructed in R from the same scLENS reduction with uwot (version 0.2.3) using cosine distance, min_dist = 0.1, and 15 nearest neighbors, with ret_model = TRUE and ret_extra = c("fgraph") used to recover both the embedding and the fuzzy simplicial set graph that was then stored as a Seurat graph object. Hierarchical and non-hierarchical datasets are separated in the corresponding per-dataset range plots (**C, D**). The stability summaries across all datasets and by structural subset (**E, F**) use the consistent clustering ratio, defined here as the number of consistent cluster numbers divided by the 19 tested values from 2 to 20. Grey points denote individual datasets, outlined bars denote the mean ratio, error bars denote mean ± 1 SD, and the numbers above the groups report datasets whose ECS-selected optimal cluster number was excluded from the corresponding consistent set. The two graph-construction strategies recovered the same optimal cluster number for 42 of 48 datasets, retained the optimal cluster number within the stable interval for 44 datasets each, and yielded nearly identical mean consistent clustering ratios, 0.3704 and 0.3698, indicating that R-native UMAP-graph reconstruction preserved the global stability landscape despite modest interval-level differences in individual datasets.

Fig. S3

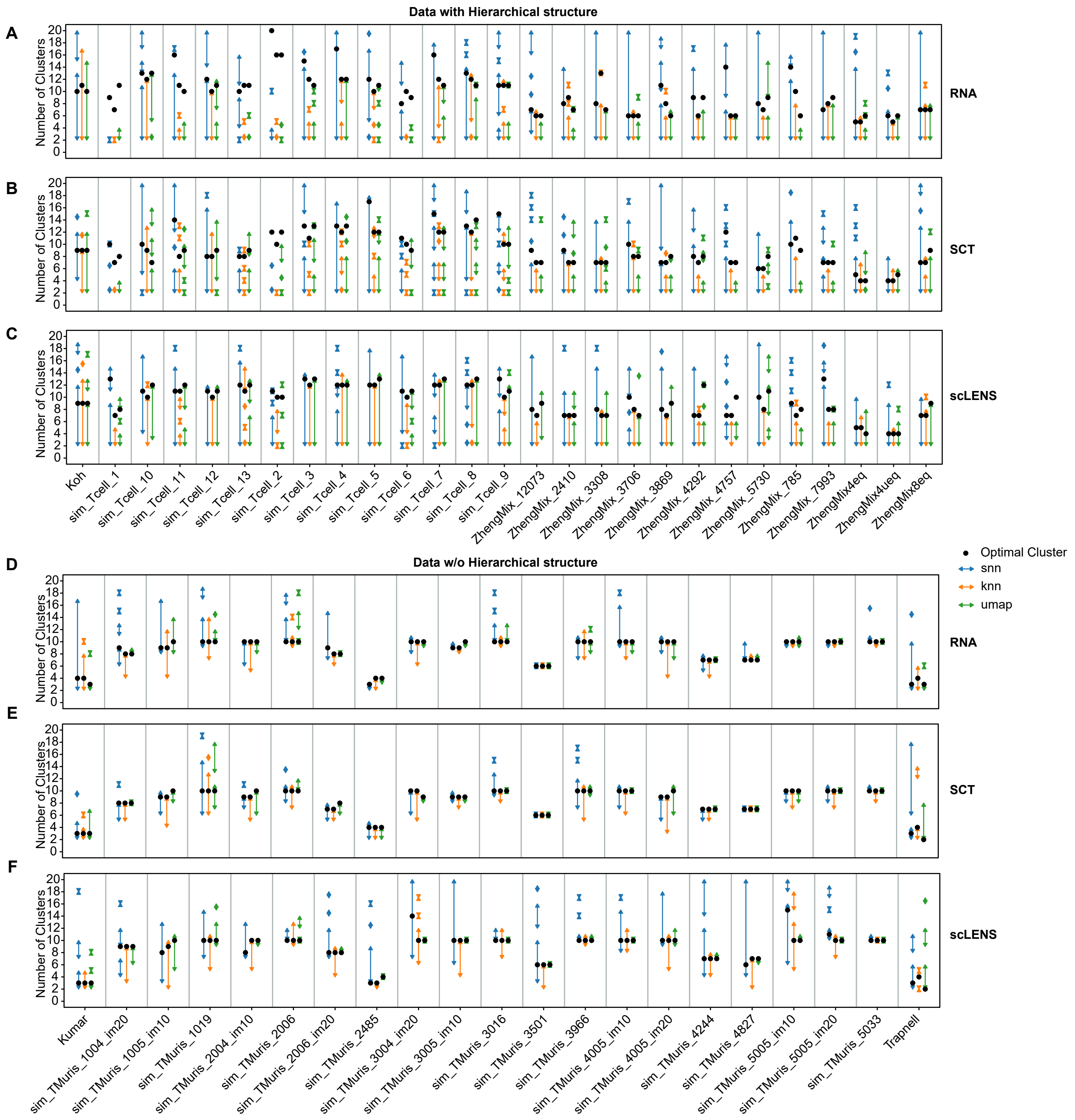

Data with Hierarchical structure
A
B
C
Number of Clusters
RNA
SCT
scLENS
Koh
sim_Tcell_1
sim_Tcell_10
sim_Tcell_11
sim_Tcell_12
sim_Tcell_13
sim_Tcell_2
sim_Tcell_3
sim_Tcell_4
sim_Tcell_5
sim_Tcell_6
sim_Tcell_7
sim_Tcell_8
sim_Tcell_9
ZhengMix_12073
ZhengMix_2410
ZhengMix_3308
ZhengMix_3706
ZhengMix_3869
ZhengMix_4292
ZhengMix_4757
ZhengMix_5730
ZhengMix_785
ZhengMix_7993
ZhengMix4eq
ZhengMix4ueq
ZhengMix8eq
Optimal Cluster
snn
knn
umap
Data w/o Hierarchical structure
D
E
F
Kumar
sim_TMuris_1004_im20
sim_TMuris_1005_im10
sim_TMuris_1019
sim_TMuris_2004_im10
sim_TMuris_2006
sim_TMuris_2006_im20
sim_TMuris_2485
sim_TMuris_3004_im20
sim_TMuris_3005_im10
sim_TMuris_3016
sim_TMuris_3501
sim_TMuris_3966
sim_TMuris_4005_im10
sim_TMuris_4005_im20
sim_TMuris_4244
sim_TMuris_4827
sim_TMuris_5005_im10
sim_TMuris_5005_im20
sim_TMuris_5033
Trapnell

Fig. S3 Effects of Preprocessing and Graph Construction on scICER Stability Assessment.

The nine workflow-graph combinations underlying the main-text summaries are expanded here at per-dataset resolution so that both the methodological meaning of each workflow and the source of the observed stability differences can be inspected directly. All combinations were benchmarked with the same target cluster range, 2 to 20, and the same logic used in Fig. S1-S2: cluster numbers with median bootstrapped IC below 1.005 formed the consistent cluster set, and the black dot marks the ECS-selected optimal cluster number against the benchmark reference labels. The three preprocessing workflows correspond to log-normalized RNA, SCTransform, and scLENS. In the RNA workflow, raw UMI counts were normalized with Seurat LogNormalize, followed by highly variable feature selection, centering and scaling, and PCA on the scaled matrix. In the SCTransform workflow, the count matrix was transformed to variance-stabilized residuals and those residuals were then subjected to PCA. In the scLENS workflow, the low-dimensional reduction obtained after L2 normalization and denoising was used directly as the input representation. Each representation was then paired with three graph construction strategies that differ conceptually as well as computationally. KNN graphs retain direct nearest-neighbor relationships, SNN graphs reweight cell-cell connections by the degree of shared neighborhood overlap and therefore tend to suppress weaker or less reproducible edges, and UMAP graphs retain the fuzzy simplicial-set connectivity learned by the low-dimensional manifold model. In practice, SNN and KNN graphs were constructed with Seurat FindNeighbors using Euclidean distance and 20 nearest neighbors, with all available dimensions in the input reduction passed to the graph-building step, whereas UMAP graphs were rebuilt with uwot using cosine distance, min_dist = 0.1, 15 nearest neighbors, and the returned fgraph object. Hierarchical datasets are ordered as RNA, SCTransform, and scLENS workflows (**A-C**), followed by the same ordering for non-hierarchical datasets (**D-F**). Within each dataset, blue, orange, and green intervals correspond to SNN-, KNN-, and UMAP-based analyses, respectively. The consistent clustering ratio summarized in the main text is the fraction of the 19 tested cluster numbers that belong to the consistent set, so broader intervals usually translate into higher ratios, although not necessarily into better biological resolution. Three patterns are apparent. First, SNN-based analyses generally yielded broader consistent sets than KNN- or UMAP-based analyses, which is compatible with the greater topological smoothing imposed by the shared-neighbor construction. Second, preprocessing and graph construction interacted strongly rather than additively. The most stable overall combination was scLENS+SNN, which reached a mean consistent clustering ratio of 0.7016 and lost the optimal cluster number from the stable set in only two datasets. RNA+SNN and SCT+SNN followed with mean ratios of 0.6495 and 0.5267 and six discordant datasets each. Third, failures accumulated predominantly in hierarchical datasets analyzed with KNN or UMAP graphs, reaching 11 and 11 discordant datasets for RNA+KNN and RNA+UMAP, 14 and 15 for SCT+KNN and SCT+UMAP, and 10 and 4 for scLENS+KNN and scLENS+UMAP. The non-hierarchical subset was uniformly less sensitive, with only one scLENS+KNN dataset placing the optimal cluster number outside the consistent set. Together, these results indicate that apparent stability is jointly determined by preprocessing, distance metric, neighborhood size, and graph formalism, and that broader stable intervals must still be interpreted against biological plausibility because conservative graph constructions can enlarge the admissible cluster-number range by repeatedly merging related cell states.

Fig. S4

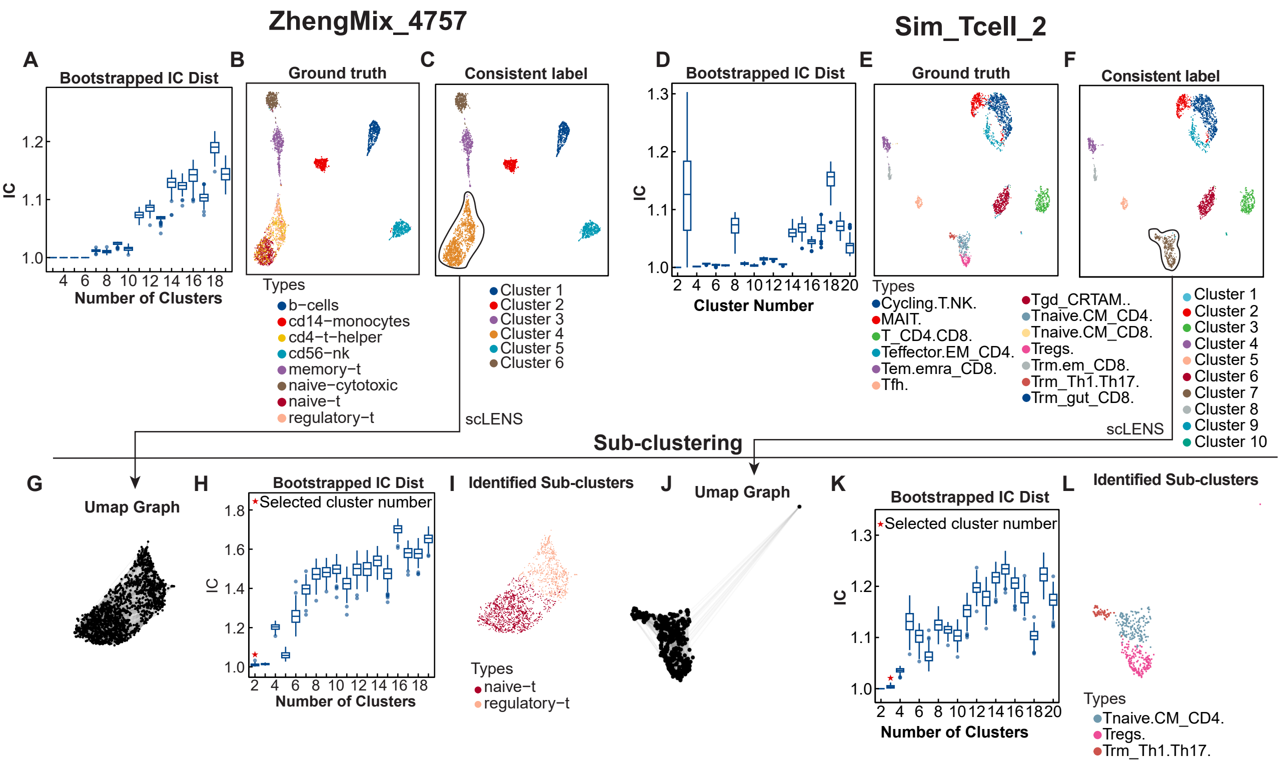

Fig. S4 Seurat-native iterative refinement of under-resolved clusters using scICER.

Globally stable solutions in ZhengMix_4757 and sim_Tcell_2 still left biologically distinct subpopulations under-resolved. The parent analysis of ZhengMix_4757 combines the bootstrapped IC distribution, the ground-truth cell-type map, and the stable scICER labels derived from the scLENS-based UMAP graph (**A-C**). Lower IC values indicate greater reproducibility across repeated Leiden trials, and the parent run used 30 Leiden trials, 200 bootstrap replicates, and seed 123. The selected global solution contained six clusters, yet comparison of the ground-truth and stable-label maps shows that naive and regulatory T cells were merged into one parent cluster. The same logic applies to sim_Tcell_2, where the parent IC distribution, ground-truth labels, and stable scICER labels (**D-F**) identify a ten-cluster solution that still merges three T-cell subtypes into one parent cluster. These cases expose the limitation of a single global resolution: the partition that is most reproducible across the full graph can still mask local heterogeneity when the dominant global structure favors merging. Iterative refinement was therefore performed by isolating the relevant parent cluster, removing previous clusters_* annotations, recomputing PCA when required, rebuilding a subset-specific UMAP graph with uwot using cosine distance, min_dist = 0.1, and n_neighbors = 15, and rerunning scICER on that local graph under the same bootstrap-based stability framework. Subset graph topology, local IC behavior, and recovered subclusters are then resolved for ZhengMix_4757 (**G-I**) and sim_Tcell_2 (**J-L**). Two stable subclusters restored the naive and regulatory T-cell separation in ZhengMix_4757, and three stable subclusters recovered the previously merged T-cell states in sim_Tcell_2. The final cluster number therefore increased from six to seven in ZhengMix_4757 and from ten to twelve in sim_Tcell_2, showing that iterative scICER can recover locally masked structure without switching to a different clustering principle or abandoning the reproducibility criterion used in the parent analysis.

Fig. S5

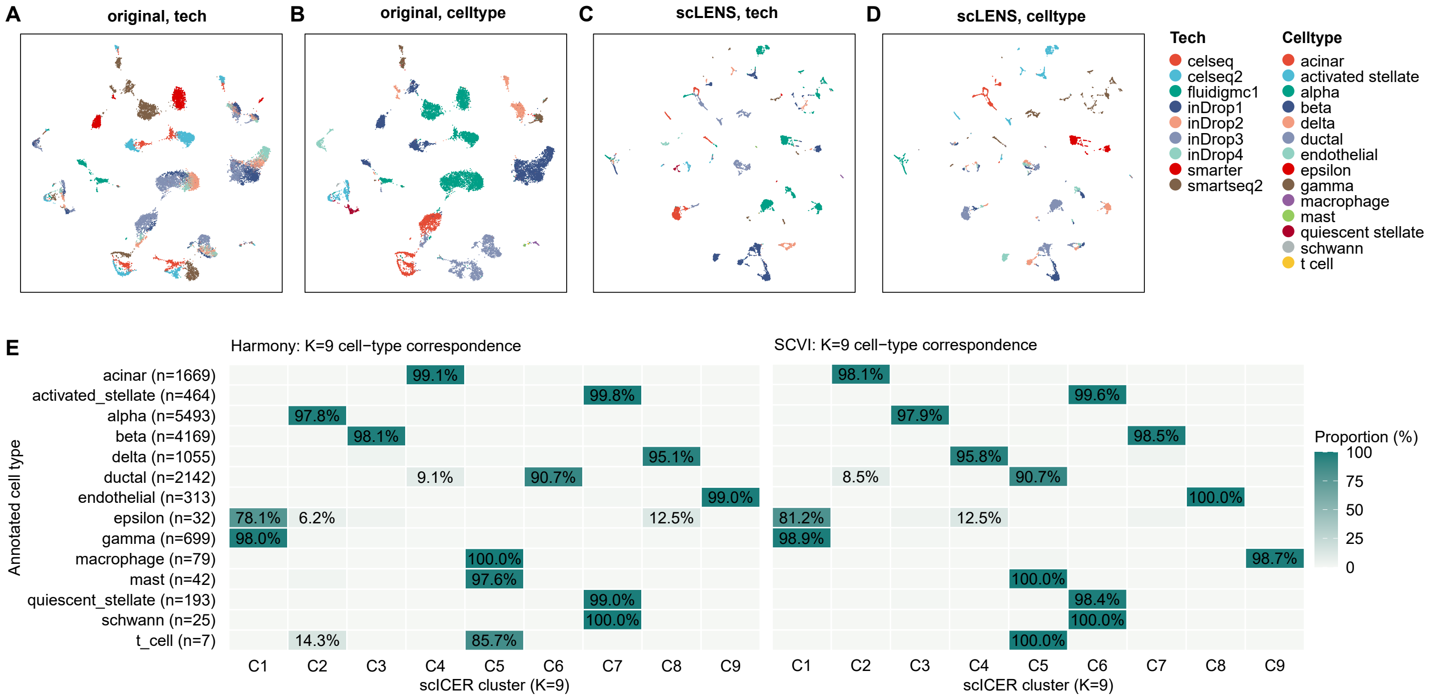

Fig. S5 Original-data and scLENS views of the benchmark human pancreas dataset and cell-type correspondence after integration.

Batch structure in the pancreas benchmark remained dominant before explicit integration. The original embeddings colored by sequencing technology and annotated cell type (**A, B**) show that cells were organized primarily by platform rather than biology. The corresponding scLENS embeddings (**C, D**) become more compact, consistent with denoising and manifold regularization, yet technology-dependent separation remains prominent and annotated cell types still fail to align as coherently as in the Harmony- and scVI-corrected embeddings in **Fig. 1F–I**. Improved compactness of the manifold was therefore not by itself sufficient to remove the batch structure in this benchmark. Following Harmony and scVI integration, cell-type-to-cluster correspondence was further examined for the scICER solutions at K = 9 (**E**). The heatmaps show the percentage of cells from each annotated cell type assigned to each scICER cluster (C1–C9), with cell counts (n) indicated in parentheses and darker colors representing higher proportions. Most annotated cell types were predominantly assigned to individual clusters, although some shared the same cluster or were distributed across multiple clusters, indicating that the K = 9 solutions captured major cell-type populations without fully resolving all annotated cell types.

Fig. S6

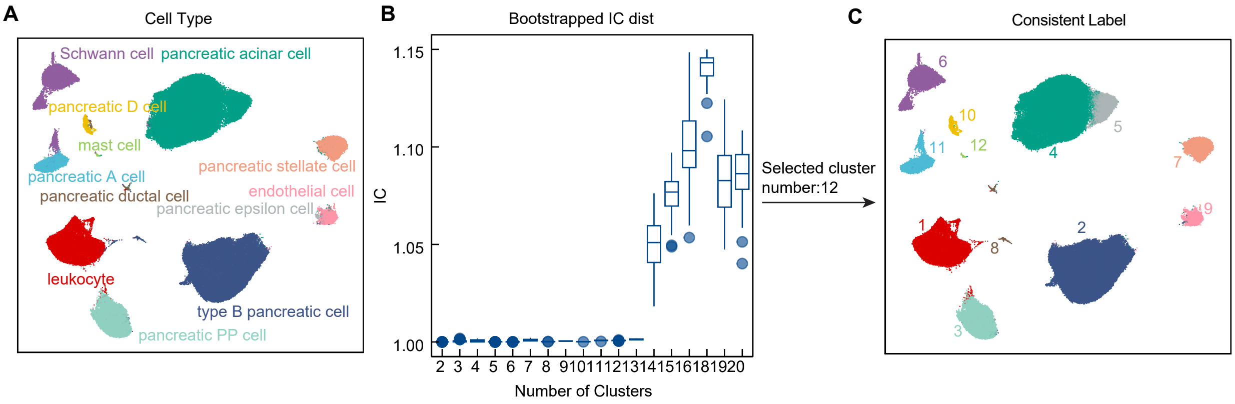

Fig. S6 Application of scICER to a large pancreatic islet atlas.

The same graph-based stability framework remained usable after standard Seurat/Harmony preprocessing in the substantially larger and more heterogeneous GSE221156 pancreatic islet atlas. The public h5ad file was converted to Seurat format, Ensembl identifiers were mapped to HGNC symbols with biomaRt, and the resulting object was normalized, scaled, reduced by PCA, and integrated with Harmony using assay and donor_id as batch-related variables. The Harmony embedding colored by annotated cell type (**A**) preserves the major endocrine, exocrine, stromal, endothelial, immune, and Schwann-cell populations after integration. scICER was then applied directly to the harmony_nn graph under the modularity objective across candidate cluster numbers from 2 to 20 using 15 repeated Leiden trials, 100 bootstrap replicates, and seed 123. The IC distribution (**B**) summarizes the reproducibility of each candidate cluster number across the bootstrap procedure, with lower values indicating that repeated stochastic clustering runs converge on the same partition more consistently. The principal trough was centered at K = 12, and the stable labels mapped back to the integrated embedding (**C**) correspond to that selected solution. Direct stability assessment on an integrated Seurat graph thus remained interpretable at a scale of 245,878 cells.

Fig. S7

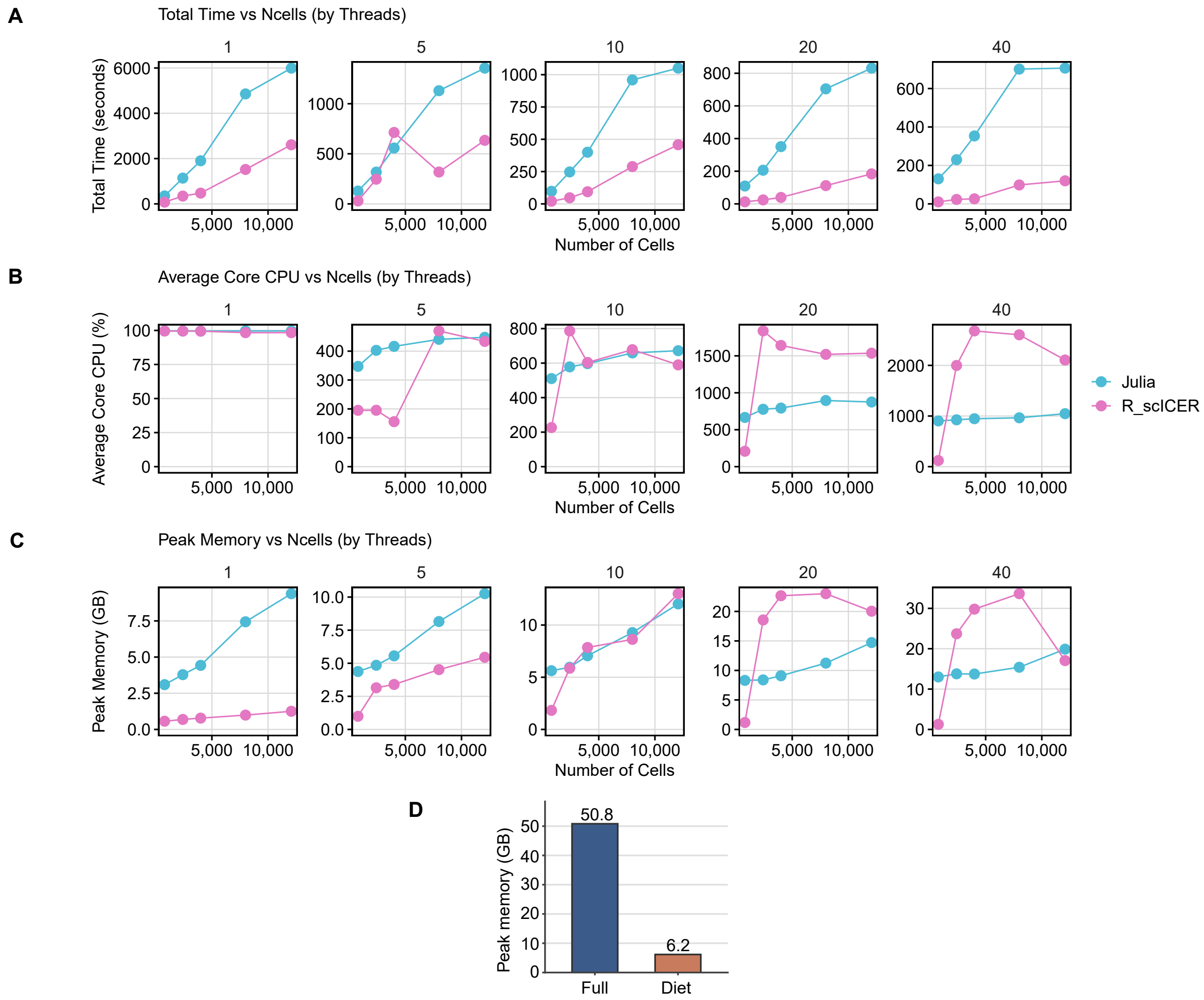

Fig. S7 Runtime, CPU utilization, and memory scaling of scICER and scICE, and memory reduction by DietSeurat-based input pruning.

Computational benchmarks across five datasets (778–11,749 cells) and five configured worker settings (1, 5, 10, 20, and 40) characterize the performance trade-offs between the Julia and Seurat-native implementations. Wall-clock runtime (**A**), average CPU utilization (**B**), and peak memory usage (**C**) were measured for scICE (cyan) and scICER (magenta). Each facet represents a fixed worker setting, allowing comparison of scaling with dataset size within facets and increasing parallelism across facets. Both implementations used their respective native workflows, with input and graph characteristics detailed in **Supplementary Table S2**. At 40 workers, scICER achieved a mean speedup of 8.17-fold across the five datasets. For the largest dataset (ZhengMix_12073), runtime decreased from 706 s to 120 s (~5.9-fold). This was accompanied by higher CPU utilization (**B**), whereas peak memory was slightly lower for scICER (17.05 versus 19.89 GB) (**C**). CPU utilization was expressed as core equivalents (100% representing one CPU core), while peak memory was defined as the maximum sampled aggregate proportional set size (PSS), accounting for shared memory across processes. Measurements were restricted to the core clustering-stability computation, excluding input loading, preprocessing, graph construction, visualization, output saving, and downstream label extraction and transfer. Memory reduction was further evaluated in the 245,878-cell GSE221156 atlas (**D**) by comparing a full Seurat object with a DietSeurat-pruned object retaining the same Harmony graph while removing most assay data. Because scICER evaluates clustering stability using graph structure and associated metadata, this reduction preserves the clustering substrate without requiring full expression matrices. At five workers and K = 12, peak memory during *scICE_clustering()* decreased from 50.8 GB to 6.2 GB (87.8%). Thus, although increased parallelism improves runtime at the cost of greater memory demand, graph-preserving input pruning can substantially alleviate this burden while allowing the resulting labels to be transferred back to the full object.
